# Ayurveda in Preventive and Supportive Healthcare: Current Evidence, Safety, and Clinical Integration

**DOI:** 10.7759/cureus.112435

**Published:** 2026-07-10

**Authors:** Jassim Rahiman K, Jyoti Prakash, Laxmi Patel, Ketki Wagh, Chhaya Trimbakrao Munde, Yogita Abhijit Jamdade

**Affiliations:** 1 Department of Kayachikitsa (Internal Medicine), All India Institute of Ayurveda, University of Delhi, New Delhi, IND; 2 Department of General Medicine, All India Institute of Medical Sciences, Patna, Patna, IND; 3 Department of Swasthavritta and Yoga, Shri Narayana Prasad Awasthi Govt. Ayurveda College, Pt. Deendayal Upadhyay Memorial Health Science and Ayush University of Chhattisgarh, Raipur, IND; 4 Department of Preventive and Social Medicine, Dr. D. Y. Patil College of Ayurved and Research Centre, Pimpri-Chinchwad, IND; 5 Department of Physiology, Dr. D. Y. Patil College of Ayurved and Research Centre, Pimpri-Chinchwad, IND; 6 Department of Samhita Siddhanta and Sanskrit, Pune District Education Association's College of Ayurveda and Research Center, Pimpri-Chinchwad, IND

**Keywords:** ayurveda, evidence-based practice, integrative medicine, lifestyle medicine, traditional medicine

## Abstract

Ayurveda represents one of the oldest structured healthcare systems and continues to gain attention within contemporary integrative medicine because of its emphasis on prevention, individualized care, lifestyle regulation, and health maintenance. Despite growing global use, its wider clinical acceptance remains limited by inconsistent evidence quality, heterogeneous formulations, variable treatment protocols, insufficient safety reporting, and challenges in aligning traditional diagnostic concepts with modern biomedical standards. This review critically examines the therapeutic relevance of Ayurveda in modern healthcare, with attention to its applications in metabolic, cardiovascular, gastrointestinal, hepatobiliary, musculoskeletal, inflammatory, mental health, respiratory, immune-related, reproductive, geriatric, and preventive care. A comprehensive narrative approach was used to synthesize evidence from clinical studies, pharmacological investigations, traditional frameworks, and integrative healthcare literature. The review indicates that specific Ayurvedic interventions, including herbal formulations, Panchakarma, Rasayana, yoga, dietary regulation, and lifestyle medicine, may offer supportive value in selected conditions through measurable outcomes such as cardiometabolic risk markers, gastrointestinal symptom scores, pain and functional indices, stress and sleep measures, respiratory symptom control, reproductive-metabolic parameters, and quality-of-life outcomes. Many claims remain insufficiently validated due to methodological weaknesses, limited standardization, and inadequate pharmacovigilance. Emerging Ayurgenomics may help bridge traditional Prakriti-based constitutional assessment with genomic profiles, molecular biomarkers, metabolic phenotypes, inflammatory signatures, and individualized risk stratification. Artificial intelligence and machine learning may further support modernization by digitizing Ayurvedic clinical records, standardizing diagnostic criteria, improving pharmacovigilance, and predicting response to polyherbal therapy. Ayurveda may contribute meaningfully to modern healthcare when applied as complementary and preventive care within regulated, evidence-based, and interdisciplinary systems. Future advancement depends on rigorous trials, validated biomarkers, quality-controlled formulations, transparent safety monitoring, and reproducible digital-clinical datasets.

## Introduction and background

Ayurveda is one of the oldest organized systems of medicine and remains relevant to current discussions on preventive, personalized, and integrative healthcare [1]. Originating in the Indian subcontinent, it views health through an interconnected framework involving the body, mind, behavior, environment, and broader well-being [1]. Its clinical orientation emphasizes health maintenance, risk reduction, restoration of physiological balance, and individualized care based on Prakriti, or a person’s constitutional profile. In contrast, conventional disease-centered care generally begins after clinical symptoms or pathology are identified and focuses on diagnosis, treatment, and disease control [2]. Core concepts such as Tridosha, Prakriti, Agni, Ama, Dhatu, and Srotas provide a systems-based framework for interpreting health and disease as dynamic processes rather than isolated biological events [3]. For readers unfamiliar with Ayurveda, these concepts should be understood as traditional clinical-reasoning categories rather than direct anatomical, biochemical, or diagnostic equivalents unless supported by empirical validation.

Complementary and integrative medicine has gained increasing global attention, encouraging further evaluation of Ayurveda within contemporary healthcare [3]. Integrative healthcare combines conventional biomedical practice with evidence-informed traditional, lifestyle, and mind-body interventions in a safe, ethical, and patient-centred manner [4]. Ayurvedic practice contributes to this model through dietary regulation, daily and seasonal routines, herbal formulations, Panchakarma, Rasayana, yoga, meditation, and behavioral modification [5]. These approaches may be relevant to chronic non-communicable disease care because they address modifiable determinants such as diet, physical inactivity, sleep disruption, stress, and long-term adherence. The translational value of Ayurveda requires disease-specific evaluation using measurable clinical outcomes, standardized interventions, and safety monitoring during concurrent use with conventional care [6].

Chronic diseases remain a major challenge for healthcare systems due to long-term medication use, high treatment costs, impaired quality of life, and limited preventive engagement [7]. Ayurveda addresses these concerns through lifestyle correction, patient participation, individualized care, and health maintenance [7]. Prakriti has been investigated as a possible model for personalized medicine because constitutional patterns may influence disease susceptibility, treatment response, and preventive strategies [8]. Similarly, Agni and Ama may be conceptually related to digestion, metabolism, inflammatory burden, and systemic imbalance; these traditional constructs should be studied scientifically without treating them as substitutes for microbiome, inflammatory, metabolic, or other biomedical markers [9]. Emerging Ayurgenomics extends this translational approach by examining whether Prakriti-based body types can be mapped to genomic profiles, molecular biomarkers, metabolic phenotypes, inflammatory signatures, microbiome patterns, and treatment-response variability [6,7].

The increasing use of Ayurvedic medicines and therapies in clinical, wellness, and commercial settings makes evidence-based evaluation necessary [9,10]. Current literature has examined Ayurvedic approaches in chronic disease prevention, metabolic and gastrointestinal disorders, inflammatory conditions, mental health support, respiratory care, women’s health, geriatric care, and rehabilitation [10]. Several medicinal plants and formulations have been investigated in experimental and clinical studies for anti-inflammatory, antioxidant, immunomodulatory, hepatoprotective, neuroprotective, and metabolic regulatory effects [11]. Specific interventions reported in the literature include Ayurvedic herbal preparations for dyslipidemia; whole-system Ayurveda protocols for irritable bowel syndrome (IBS); curcumin and turmeric extracts for arthritis-related outcomes; Ashwagandha for stress and anxiety; Ayurvedic interventions for allergic rhinitis; and lifestyle-based approaches for polycystic ovary syndrome (PCOS)-related metabolic and reproductive outcomes [12,13]. These pharmacological and clinical findings may support biological plausibility, but therapeutic claims require confirmation through robust trial design, defined formulations, appropriate comparators, validated outcomes, adequate follow-up, and systematic adverse-event reporting.

Although Ayurveda is widely used, its integration into modern healthcare remains limited by evidence heterogeneity and safety concerns. Several clinical studies, including randomized controlled trials, observational studies, pilot studies, and pragmatic evaluations, have examined Ayurvedic interventions in chronic disease and supportive care contexts; translational confidence remains limited by small sample sizes, inadequate or unclear randomization, limited blinding, short follow-up, heterogeneous interventions, variable outcome measures, and inconsistent adverse-event reporting [11]. Additional concerns include variability in raw materials, contamination risk, heavy metal exposure, differences between classical and commercial preparations, adulteration, dose inconsistency, herb-drug interactions, and inadequate pharmacovigilance [14]. These issues are clinically important when Ayurvedic medicines are used alongside conventional drugs because interactions may affect efficacy or patient safety. Artificial intelligence and machine learning may help address these limitations by digitizing Ayurvedic clinical records, structuring Prakriti and symptom data, standardizing diagnostic criteria, detecting adverse-event patterns, supporting quality-control analytics, and predicting individualized response to polyherbal therapy [4,8,12,15].

This review applies a thematic narrative synthesis rather than a formal, systematic, or quantitative analytical method. The analysis is organized around predefined domains: core Ayurvedic concepts, evidence from clinical and pharmacological studies, therapeutic applications in major chronic disease areas, proposed mechanisms, safety concerns, quality-control issues, Ayurgenomics, artificial intelligence, machine learning, digital standardization, and barriers to integration. Within each domain, the available evidence is interpreted by considering clinical relevance, consistency of findings, methodological quality, intervention standardization, outcome measures, adverse-event reporting, and translational applicability. This review therefore focuses on Ayurveda as a preventive, complementary, and supportive approach rather than as a substitute for evidence-based conventional diagnosis or treatment. It further identifies research priorities, including rigorous clinical trials, validated biomarkers, standardized interventions, quality-controlled formulations, pharmacovigilance, digitized clinical datasets, molecular phenotyping, and interdisciplinary care models.

Objective of the review

This review aims to critically evaluate the role of Ayurveda in contemporary healthcare as a preventive, complementary, and supportive approach, with particular attention to clinical applications, evidence quality, safety, toxicity concerns, and integration with conventional care. It specifically examines issues relevant to clinical translation, including heavy metal contamination, adulteration, variability in herbal formulations, dose-related adverse effects, herb-drug interactions, inadequate pharmacovigilance, quality-controlled manufacturing, and systematic adverse-event reporting. The review also examines specific Ayurvedic and Ayurveda-related therapeutic interventions with reported or measurable outcomes across major disease areas, including metabolic, gastrointestinal, musculoskeletal, mental health, respiratory, reproductive, and geriatric care. It further discusses Ayurgenomics, artificial intelligence, and machine learning as emerging tools for mapping Prakriti to molecular biomarkers, digitizing clinical records, standardizing diagnostic criteria, improving pharmacovigilance, and predicting response to polyherbal therapy.

## Review

Methodology

This review was prepared as a thematic narrative synthesis with a critical appraisal of published literature on Ayurveda in contemporary healthcare. As this article was designed as a narrative review rather than a systematic review or meta-analysis, PRISMA reporting was not applied; instead, the review used predefined thematic domains and a consistent critical appraisal framework to organize and interpret the literature. No statistical pooling, meta-analysis, or meta-regression was performed because the review was designed as a narrative synthesis, and the included literature was heterogeneous in study design, intervention type, comparator selection, outcome measures, follow-up duration, and safety reporting. Formal study-level risk-of-bias scoring was not performed because this was a narrative review; methodological limitations such as sample size, randomization, blinding, comparator selection, follow-up duration, outcome validity, and adverse-event reporting were critically considered during evidence interpretation.

Searches were conducted in PubMed, Scopus, Web of Science, Google Scholar, the Cochrane Library, AYUSH-related sources, and indexed journal platforms. The search terms included Ayurveda, integrative medicine, evidence-based Ayurveda, Ayurvedic clinical trials, Panchakarma, Rasayana, herbal medicine, chronic disease, metabolic disorders, gastrointestinal disorders, musculoskeletal disease, mental health, respiratory disorders, personalized medicine, Ayurgenomics, Prakriti, genomics, molecular biomarkers, artificial intelligence, machine learning, digital health records, diagnostic standardization, polyherbal therapy response prediction, systems biology, safety, toxicity, pharmacovigilance, herb-drug interactions, and quality control. Literature published from 2018 to 2026 was prioritized, while older sources were used only when necessary to explain classical Ayurvedic principles or foundational concepts. Reference selection emphasized recent, clinically relevant, and topic-specific literature, and each source was used only where it directly supported the corresponding conceptual, clinical, safety, digital, molecular, or translational discussion.

Eligible sources included randomized controlled trials, observational studies, pragmatic clinical studies, pilot studies, systematic reviews, meta-analyses, pharmacological studies, Ayurgenomics studies, digital-health studies, artificial intelligence and machine-learning discussions relevant to Ayurveda, and peer-reviewed narrative reviews. Studies and reviews were considered when they addressed Ayurvedic principles, therapeutic applications, clinical outcomes, safety, toxicity, quality control, pharmacovigilance, Prakriti-based molecular profiling, diagnostic standardization, clinical record digitization, or integration with conventional healthcare. Non-peer-reviewed items, unsupported claims, duplicate records, inaccessible full-text articles, publications without clear relevance to Ayurveda, and poorly described studies lacking defined interventions or outcomes were excluded.

The included literature was organized into predefined thematic domains, including conceptual foundations, clinical applications, evidence quality, proposed mechanisms, safety and toxicity concerns, quality-control issues, therapeutic interventions, reported clinical outcomes, Ayurgenomics, artificial intelligence, machine learning, digital standardization, and barriers to integration. Each domain was interpreted using a consistent evaluative framework that considered clinical relevance, study design, intervention standardization, comparator use, outcome validity, follow-up duration, adverse-event reporting, and translational applicability. For therapeutic sections, emphasis was placed on identifying specific interventions, target disease areas, reported or measurable outcomes, and limitations affecting clinical interpretation. For digital and molecular sections, emphasis was placed on the potential of Prakriti-biomarker mapping, structured clinical datasets, diagnostic standardization, pharmacovigilance, and prediction of individualized response to polyherbal therapy. Conclusions were drawn cautiously according to the strength, consistency, and clinical applicability of the available evidence rather than from traditional use or biological plausibility alone.

Principles and conceptual foundations of Ayurveda

Ayurveda defines health as a dynamic state shaped by interactions among the body, mind, behavior, environment, and consciousness [13]. Its conceptual framework is built on core principles such as Tridosha, Prakriti, Agni, Ama, Dhatu, Srotas, and Ojas, which are used to interpret constitution, digestion-metabolism, tissue nourishment, physiological transport, disease development, and resilience [13]. Tridosha describes three functional regulatory principles: Vata, related to movement and communication; Pitta, related to transformation and metabolism; and Kapha, related to structure and stability. These principles are best interpreted as traditional functional categories that guide clinical reasoning, disease-pattern recognition, and individualized treatment selection rather than as direct biomedical mechanisms [14].

Individualization in Ayurveda is mainly based on Prakriti, which classifies individuals according to constitutional patterns that may influence disease susceptibility, clinical presentation, preventive needs, and response to treatment. This idea has attracted interest in relation to personalized medicine because both approaches recognize interindividual variation in health risks and therapeutic responses [15]. Emerging Ayurgenomics extends this concept by examining whether classical Prakriti types can be mapped to genomic profiles, molecular biomarkers, metabolic signatures, inflammatory patterns, microbiome features, and drug-response variability [6,7,15]. The concepts of Agni and Ama are also used to explain digestive-metabolic function and the accumulation of incompletely processed biological material. These constructs may be studied in relation to metabolism, gut function, inflammation, and systemic imbalance, but their clinical use requires validated biomarkers and reproducible diagnostic criteria [16].

Prevention is another major component of Ayurveda and includes daily routine, seasonal adaptation, dietary regulation, sleep hygiene, mental discipline, and support of Ojas, which is traditionally associated with resilience and vitality. This preventive orientation overlaps with contemporary lifestyle medicine and public health strategies that target chronic disease risk reduction [17]. Its clinical value depends on whether specific Ayurvedic interventions, such as dietary regulation, Rasayana, Panchakarma, yoga, herbal formulations, and behavioral modification, produce measurable outcomes in defined disease contexts, including metabolic markers, gastrointestinal symptoms, pain scores, stress measures, respiratory outcomes, reproductive-metabolic parameters, and quality of life. Several Ayurvedic constructs remain difficult to evaluate using current biomedical tools because definitions, diagnostic criteria, and measurable biomarkers are not yet sufficiently standardized. Digital records, standardized clinical descriptors, and biomarker-linked phenotyping may help make these traditional assessments more reproducible, while the detailed role of artificial intelligence and machine learning is discussed separately in the dedicated digital-standardization section. The major Ayurvedic concepts, including Tridosha, Prakriti, Agni, Ama, Dhatu, Upadhatu, Mala, Srotas, and Ojas, and their possible biomedical interpretations are summarized in Table 1.

**Table 1:** Core Ayurvedic Concepts and Possible Biomedical Correlates

Ayurvedic Concept	Traditional Interpretation	Possible Biomedical Correlate	Clinical Relevance	Reference
Tridosha	Functional principles governing movement, transformation, and structure through Vata, Pitta, and Kapha	Regulatory physiology, neuroendocrine balance, metabolic regulation, homeostasis	Used for diagnosis, constitutional assessment, disease interpretation, and treatment planning; requires standardized clinical descriptors to improve reproducibility across practitioners	[1,13]
Prakriti	An individual’s constitutional pattern is determined by inherited and acquired factors	Personalized medicine, phenotypic profiling, genetic and metabolic variability; emerging Ayurgenomics may map Prakriti types to genomic profiles, molecular biomarkers, metabolic signatures, inflammatory patterns, and microbiome features	May guide preventive strategies, disease risk assessment, individualized therapy, biomarker-based stratification, and prediction of treatment response	[6,15]
Agni	Digestive and metabolic capacity is responsible for the transformation of food and tissue nourishment	Digestion, metabolism, enzymatic activity, gut function, metabolic efficiency; potentially measurable through metabolic, inflammatory, gut-function, and microbiome-related markers	Relevant to gastrointestinal disorders, metabolic disease, nutritional status, and systemic health; requires validated outcome measures rather than symptom-based interpretation alone	[1,7]
Ama	Improperly processed biological material is associated with obstruction and disease progression	Metabolic waste, inflammatory burden, dysbiosis-related dysfunction, and toxic metabolic intermediates may be explored through inflammatory biomarkers, metabolomics, and gut-microbiome assessment	Used to explain chronic inflammation, impaired metabolism, and systemic imbalance; AI-supported structured symptom recording may help standardize assessment	[7,12]
Dhatu	Tissue systems that sustain structure and function	Tissue physiology, nutritional status, musculoskeletal and immune integrity	Relevant to chronic disease, degeneration, reproductive health, and aging, clinical translation requires functional, nutritional, reproductive, and geriatric outcome measures	[1]
Upadhatu	Secondary tissue derivatives formed during Dhatu metabolism, traditionally including structures such as Rasa-derived breast milk and menstrual fluid, Rakta-derived vessels and tendons, and other supportive tissue derivatives	Tissue derivatives, accessory structural components, reproductive and vascular-supportive tissues, and secondary products of tissue metabolism	Relevant to reproductive health, lactation, menstrual physiology, vascular integrity, tissue repair, and interpretation of tissue-specific imbalance; outcomes should include menstrual, reproductive, vascular, and tissue-repair parameters where applicable	[1,13]
Mala	Physiological waste products generated through digestion and tissue metabolism, classically including Purisha, Mutra, and Sveda	Excretory products, bowel elimination, urine output, sweat regulation, detoxification pathways, and metabolic clearance	Relevant to gastrointestinal function, urinary regulation, fluid balance, metabolic waste clearance, and assessment of digestive and systemic health; should be assessed using measurable bowel, urinary, hydration, and metabolic-clearance indicators	[1,13]
Srotas	Physiological channels responsible for transport and circulation	Circulatory, lymphatic, gastrointestinal, respiratory, and metabolic pathways	Used to understand obstruction, impaired transport, and organ-system dysfunction; digital records and machine-learning models may help link Srotas-based patterns with clinical, imaging, laboratory, and functional datasets	[1,15]
Ojas	Vital essence associated with resilience, immunity, and vitality	Immune competence, stress resilience, and physiological reserve, potentially explored through immune markers, stress biomarkers, frailty measures, and quality-of-life indices	Relevant to prevention, immune support, geriatric care, and Rasayana therapy; requires measurable endpoints rather than broad claims of immunity or rejuvenation	[11,18]

Ayurvedaand evidence-based medicine

Traditional Ayurvedic knowledge is increasingly being evaluated through evidence-based methods to determine whether its interventions are effective, safe, reproducible, and clinically relevant [16]. This transition does not require rejection of classical principles; rather, it requires that Ayurvedic concepts, formulations, procedures, and lifestyle interventions be assessed using appropriate clinical and scientific methods. These methods include randomized controlled trials, observational studies, pragmatic studies, pharmacological investigations, systematic reviews, meta-analyses, and real-world evidence studies [17]. Evidence-based evaluation should also include disease-specific outcomes, such as lipid parameters in dyslipidemia, bowel-symptom scores in IBS, pain and function scores in arthritis, stress and anxiety scales, respiratory symptom scores, reproductive-metabolic outcomes in PCOS, and geriatric functional measures.

Evidence-based Ayurveda should distinguish between biological plausibility, traditional use, and clinically proven benefit. Several Ayurvedic botanicals, compound formulations, Panchakarma-based procedures, Rasayana therapies, and lifestyle interventions have been studied in metabolic, inflammatory, musculoskeletal, stress-related, and preventive care contexts [2,16]. Examples include Ayurvedic herbal preparations for hypercholesterolemia and dyslipidemia, whole-system Ayurveda protocols for IBS, curcumin and turmeric extracts for arthritis and knee osteoarthritis, Ashwagandha for stress and anxiety, Ayurvedic interventions for allergic rhinitis, and lifestyle or botanical approaches relevant to PCOS-related metabolic outcomes [19,20]. Some studies report improvements in clinical symptoms, inflammatory markers, metabolic parameters, pain scores, quality of life, or patient-reported outcomes. Other investigations describe antioxidant, anti-inflammatory, immunomodulatory, hepatoprotective, neuroprotective, and metabolic regulatory properties of commonly used Ayurvedic plants and formulations [1,18]. These findings support further investigation, particularly because many Ayurvedic formulations are multi-component and may act through multiple biological pathways. Ayurgenomics may strengthen this evaluation by linking Prakriti-based phenotypes with genomic profiles, molecular biomarkers, metabolic signatures, inflammatory patterns, microbiome characteristics, and treatment-response variability [6,7,15].

The current evidence base remains inconsistent, and positive findings should be interpreted cautiously. Many studies are limited by small sample sizes, weak comparator groups, inadequate randomization, limited blinding, short follow-up, variable outcome measures, and incomplete adverse-event reporting [17,18]. Additional translational barriers include formulation variability, uncertain dose-response relationships, practitioner-dependent protocols, limited quality-control data, inconsistent diagnostic standardization, and difficulty separating the effect of individual components in multimodal interventions. Digital clinical records, biomarker-linked phenotyping, and standardized diagnostic documentation may help improve reproducibility, while the detailed role of artificial intelligence and machine learning is addressed in the dedicated digital-standardization section. Future evidence-based Ayurveda requires well-designed clinical trials, standardized reporting, quality-controlled products, validated outcome measures, longer safety follow-up, pharmacovigilance systems, biomarker-linked phenotyping, externally validated predictive models, and transparent documentation of both benefits and harms.

Therapeutic interventions and reported clinical outcomes

Evidence on Ayurveda remains heterogeneous, but several disease-focused interventions have been evaluated in clinical or translational contexts. In metabolic and cardiovascular disorders, Ayurvedic and herbal interventions have mainly targeted dyslipidemia, obesity, metabolic syndrome, and impaired glucose regulation. Herbal medicines used for dyslipidemia have been reviewed across meta-analyses, with findings suggesting possible lipid-modifying effects, although the evidence remains limited by variation in formulation, dose, comparator selection, duration, and methodological quality [19]. Contemporary reviews of Ayurveda in healthcare have also described selected therapeutic interventions for chronic metabolic conditions, including lifestyle regulation, dietary correction, herbal formulations, yoga, and integrative care models [20]. Ayurvedic interpretations of obesity have been linked with Meda Dhatu imbalance and metabolic dysfunction, although these traditional constructs require biomedical validation before being used as clinical markers [21]. Swasthavritta and yoga-based approaches have been discussed for metabolic syndrome, with expected outcomes including lifestyle adherence, weight control, physical activity, stress regulation, and cardiometabolic risk reduction [22]. In a pilot clinical trial, *Tinospora cordifolia* attenuated metabolic alterations in hypertriglyceridemia, suggesting a possible supportive role in lipid regulation when used with dietary and medical supervision [23]. Clinically relevant outcomes in future metabolic studies should include fasting glucose, HbA1c, triglycerides, total cholesterol, low-density lipoprotein (LDL) cholesterol, high-density lipoprotein (HDL) cholesterol, body mass index, waist circumference, blood pressure, medication use, and adverse events. These cardiometabolic data illustrate the need to link each intervention to defined outcomes rather than presenting Ayurveda only as a modernization priority.

In gastrointestinal disorders, whole-system Ayurveda has been evaluated in IBS using a randomized controlled clinical trial design [24]. Herbal medicines have also been discussed for the modulation of gut microbiota, digestive symptoms, and metabolic-gastrointestinal interactions [25]. Complementary and alternative medicine approaches for IBS have been reviewed, with outcomes commonly including abdominal pain, bloating, bowel frequency, stool form, global symptom severity, rescue medication use, and quality of life [26]. The microbiome has also been discussed from both modern medical and Ayurvedic perspectives, supporting further investigation of gut-related mechanisms in digestive and systemic health [27]. Traditional Indian medicine has additionally been discussed in relation to microbiome interactions and the gut-lung axis, indicating that future studies should use objective microbial, inflammatory, respiratory, and gastrointestinal endpoints rather than broad explanatory claims [28]. These interventions should be interpreted as supportive measures after exclusion of alarm features and should not replace conventional diagnostic evaluation when red-flag symptoms are present. Future gastrointestinal research should use structured records to capture Agni-related symptoms, bowel patterns, dietary triggers, formulations, co-medications, adverse events, and patient-reported outcomes in a standardized format.

In musculoskeletal and inflammatory disorders, Ayurvedic care commonly includes internal herbal formulations, external oil therapies, fomentation, Panchakarma-related procedures, anti-inflammatory botanicals, diet correction, and rehabilitation. Curcumin and *Curcuma longa* extract have been evaluated in randomized controlled trials and meta-analyses for arthritis-related conditions, with reported benefits in pain, stiffness, inflammatory symptoms, and functional outcomes [29]. Turmeric extracts have also been studied in knee osteoarthritis, where clinically relevant outcomes include pain reduction, physical function, stiffness, analgesic use, and safety [30]. Rheumatoid arthritis-related literature has discussed herbal medicine-based therapeutic implications, although modern rheumatologic diagnosis, disease activity assessment, imaging, and serological evaluation remain essential [31]. Ayurvedic interventions in rheumatoid arthritis have also been considered in systematic-review protocols, reflecting the need for more rigorous evidence synthesis and standardized outcome assessment [32]. Future trials should assess visual analogue scale or numerical pain scores, Western Ontario and McMaster Universities Osteoarthritis Index (WOMAC) score, Disease Activity Score in 28 joints (DAS28), range of motion, inflammatory markers, analgesic consumption, function, quality of life, and adverse events. Prakriti assessment, inflammatory biomarkers, imaging findings, treatment composition, and follow-up response may also help identify subgroups more likely to benefit from specific polyherbal or procedural interventions.

In mental health and stress-related conditions, *Withania somnifera *has been evaluated in randomized and systematic review evidence. A double-blind randomized placebo-controlled clinical study reported adaptogenic and anxiolytic effects of Ashwagandha root extract in healthy adults [33]. Another randomized double-blind placebo-controlled study evaluated the stress-relieving and pharmacological actions of Ashwagandha extract [34]. A systematic review and meta-analysis further examined Ashwagandha for stress and anxiety outcomes [35]. A more recent systematic review and dose-response meta-analysis also evaluated the effects of Ashwagandha on adult mental health outcomes [36]. Measurable outcomes in this field include perceived stress scores, anxiety scales, sleep quality, cortisol-related markers, quality-of-life indices, and adverse events. These findings support a possible adjunctive role in stress regulation and general well-being, but they should not be presented as evidence for replacing psychiatric, neurological, or sleep-medicine care [37]. Longitudinal symptom scales and patient-reported outcome systems may help distinguish general well-being effects from clinically meaningful changes in stress, anxiety, sleep, and functional status.

In respiratory and immune-related conditions, Ayurvedic and yoga-based interventions have been studied or proposed for respiratory symptom support, allergic rhinitis, and post-viral recovery. Ayurveda-based intervention plans were discussed during the COVID-19 pandemic, mainly as pragmatic supportive approaches rather than replacements for acute medical care [37]. Ayurvedic interventions and yoga have also been evaluated in a structured randomized trial protocol for long-term effects after COVID-19, showing the importance of measurable safety and efficacy endpoints [38]. In allergic rhinitis, a multicentre open-label clinical study evaluated Ayurvedic interventions with outcomes relevant to symptom control and patient-reported improvement [39]. A case-based report also described Ayurvedic therapeutics for allergic rhinitis interpreted through the Vata-Kaphaja Pratishyaya framework [40]. Respiratory and immune-related claims should use measurable endpoints rather than broad terms such as “immune boosting.” Appropriate outcomes include allergic rhinitis symptom score, asthma control score, pulmonary function tests, rescue medication use, exacerbation frequency, infection frequency, inflammatory markers, quality of life, adverse events, and herb-drug interaction monitoring. Routine integrative-care records should also document interactions with bronchodilators, corticosteroids, antihistamines, and other respiratory medications.

In women’s health, especially PCOS, evidence should be framed carefully because not all available studies are Ayurveda-specific. Lifestyle and movement-based interventions have demonstrated relevance in PCOS, including diet, physical activity, weight control, metabolic risk reduction, and behavioral support. A randomized controlled trial of Tai Chi combined with dietary intervention reported effects on health-promoting lifestyle and metabolic and reproductive outcomes in women with PCOS [41]. Combined nutritional and pharmacological interventions have also been evaluated in PCOS, with outcomes related to metabolic and reproductive parameters [42]. Licorice extract combined with a low-calorie diet has been studied in overweight or obese women with PCOS, using obesity indices, glycemic indices, and lipid profiles as measurable outcomes [43]. Lifestyle management in PCOS extends beyond diet and physical activity and includes behavioral, metabolic, reproductive, and long-term adherence considerations [44]. These studies support the broader relevance of lifestyle and selected botanical approaches, but Ayurveda-specific claims require direct Ayurvedic trials with clearly defined formulations and protocols. Relevant endpoints include menstrual regularity, ovulation status, androgen profile, insulin resistance markers, body mass index, waist circumference, fertility outcomes, menopausal symptom scores, pregnancy safety, quality of life, and adverse events. Future Ayurveda-specific studies in PCOS should record Prakriti, menstrual phenotype, metabolic biomarkers, formulation composition, dose, adherence, and reproductive outcomes to support response-pattern analysis.

Across these disease areas, Ayurveda is most defensible as adjunctive and supportive care. Claims should be linked to specific interventions, defined patient populations, measurable outcomes, and transparent safety reporting rather than generalized statements about modernization or traditional use alone [1-5,17,18]. Digital standardization, Prakriti-biomarker mapping, pharmacovigilance, and response-prediction models are discussed in detail in the dedicated Ayurgenomics and digital-standardization section. Current evidence supports cautious clinical exploration of Ayurveda in chronic disease support, functional symptom management, stress regulation, lifestyle modification, and patient-centred care, but stronger conclusions require standardized formulations, reproducible protocols, appropriate comparators, validated endpoints, longer follow-up, and systematic adverse-event monitoring.

Therapeutic applications in metabolic and cardiovascular disorders

The therapeutic sections follow a consistent structure by outlining the Ayurvedic explanatory framework, summarizing contemporary evidence, and identifying limitations, safety concerns, and measurable endpoints. This structure distinguishes conceptual parallels from established clinical or mechanistic evidence and reduces redundancy by keeping broader issues of modernization, standardization, and safety in the dedicated evidence-based Ayurveda, Ayurgenomics, and future-directions sections.

Metabolic and cardiovascular disorders are relevant because their modifiable determinants, including diet, physical inactivity, adiposity, sleep disturbance, and stress, are also addressed in Ayurvedic lifestyle-based care. In Ayurvedic interpretation, diabetes mellitus and related metabolic dysfunction are discussed through disturbances in Agni, accumulation of Ama, derangement of Kapha, Meda dhatu involvement, and impaired circulatory regulation [1,7,21]. These concepts should be viewed as traditional explanatory frameworks rather than direct equivalents of insulin resistance, dyslipidemia, endothelial dysfunction, or inflammatory biomarkers. Ayurgenomics may help examine whether Prakriti patterns and Meda-related phenotypes correlate with genomic variation, lipid metabolism, inflammatory markers, adiposity indices, gut-microbiome profiles, and cardiometabolic risk signatures [6,7].

In diabetes mellitus, metabolic syndrome, obesity, and dyslipidemia, Ayurvedic care is best described as a supportive lifestyle-oriented approach rather than a replacement for evidence-based cardiometabolic management. Its proposed role includes dietary regulation, weight control, physical activity, stress reduction, sleep regulation, and selected herbal or behavioral interventions under appropriate clinical supervision. Relevant outcomes include fasting glucose, HbA1c, lipid profile, waist circumference, body mass index, blood pressure, medication use, adherence, adverse events, and herb-drug interactions. Botanicals such as *Gymnema sylvestre*,* Trigonella foenum-graecum*, *C. longa*, *Emblica officinalis*, and *T. cordifolia* have been investigated for glucose-related, antioxidant, anti-inflammatory, and lipid-modulating effects, but their relevance depends on dose standardization, formulation quality, comparator design, safety monitoring, and reproducibility [20]. A pilot clinical trial of *T. cordifolia* in hypertriglyceridemia further supports the need to report intervention-specific outcomes rather than broad claims of metabolic correction [23].

**Figure 1:**
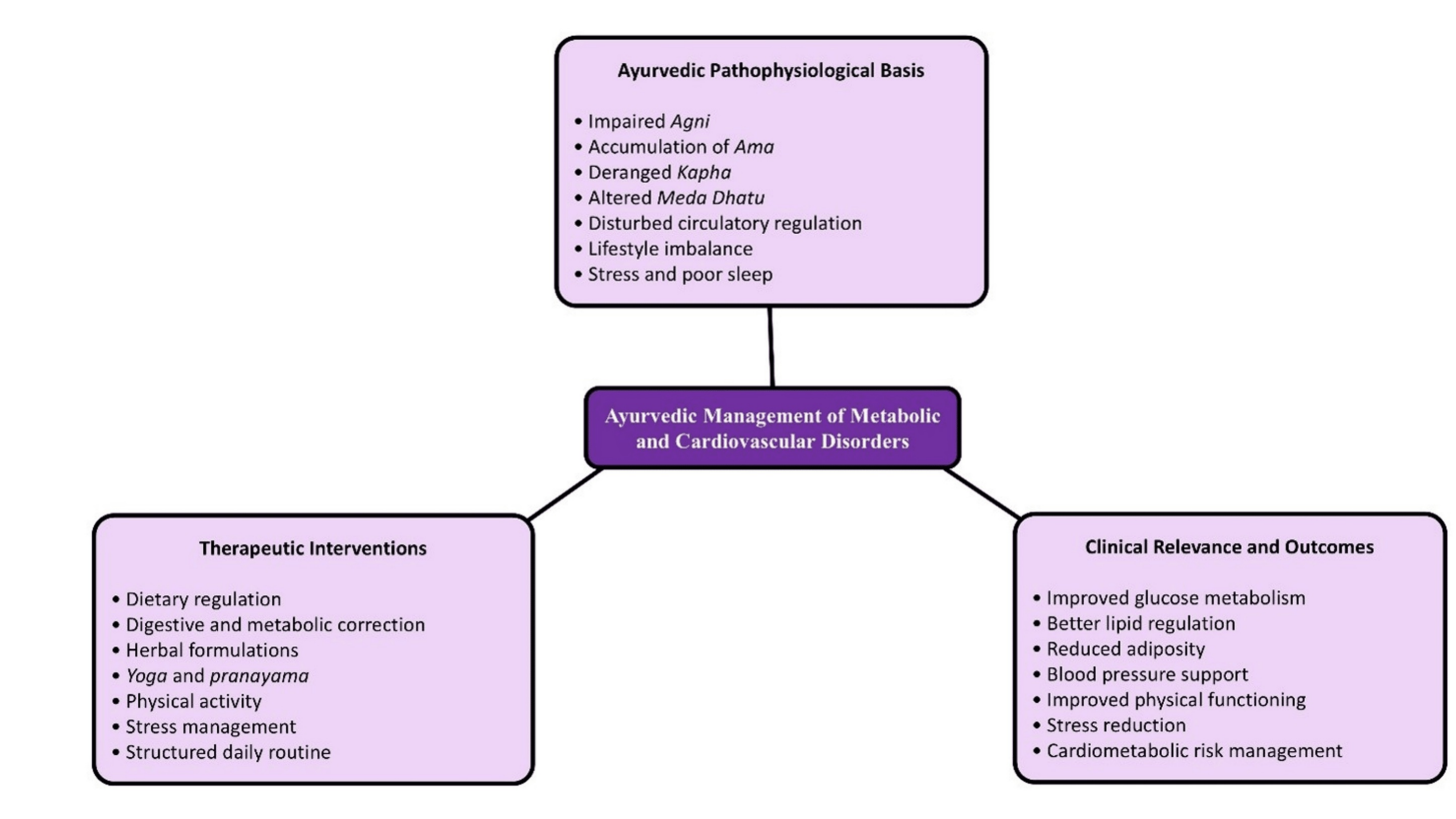
Ayurvedic Management of Metabolic and Cardiovascular Disorders Image created by the authors using Microsoft PowerPoint (Microsoft Corp., Redmond, WA, USA).

The main analytical challenge is that Ayurvedic cardiometabolic care is usually multimodal, making it difficult to isolate the effects of herbs, diet, yoga, counseling, weight reduction, sleep regulation, and adherence. Future studies should use predefined protocols, appropriate comparators, adherence monitoring, standardized clinical records, biomarker-based outcomes, longer follow-up, and documentation of Prakriti, Agni, Ama, Meda-related features, intervention details, safety events, and biomedical outcomes. Larger trials with externally validated predictive models are required before stronger clinical claims can be made. Figure 1 summarizes the proposed supportive role of Ayurvedic care in cardiometabolic disorders.

Applications in gastrointestinal and hepatobiliary disorders

Gastrointestinal function is central to Ayurvedic clinical reasoning because digestion, assimilation, elimination, and tissue nourishment are considered key determinants of health. Functional gastrointestinal symptoms and hepatobiliary disorders are traditionally interpreted through impaired Agni, accumulation of Ama, disturbance of Vata, altered bowel regulation, and defective nutrient transformation [1,7,24]. These constructs may explain Ayurvedic treatment selection, but they should not be equated directly with IBS, functional dyspepsia, constipation, dysbiosis, hepatic steatosis, or gut-liver axis biomarkers without empirical validation. Ayurgenomics and microbiome-oriented research may help clarify whether Agni- and Ama-related phenotypes correspond to reproducible microbial, inflammatory, metabolomic, or gut-liver axis signatures [6,7,27].

Ayurvedic care for functional gastrointestinal symptoms is typically individualized and may include dietary adjustment, meal timing, bowel routine formation, digestive or carminative herbs, selected oil-based preparations, mild bowel-regulating measures, and lifestyle correction [24]. Herbs such as *Zingiber officinale*, *Piper longum*, *Terminalia chebula*, *Aegle marmelos*, and *Foeniculum vulgare* have been traditionally used for carminative effects, bowel regulation, and abdominal symptom relief [25]. Whole-system Ayurveda has also been evaluated in IBS, where clinically relevant outcomes include abdominal pain, bloating, bowel frequency, stool form, global symptom severity, rescue medication use, and quality of life [24,26]. For constipation and functional gastrointestinal symptoms, Ayurvedic care should be described as supportive rather than as a replacement for dietary fibre, hydration, physical activity, clinically indicated laxatives, or diagnostic evaluation when alarm features are present.

For hepatobiliary disorders, particularly non-alcoholic fatty liver disease (NAFLD), Ayurveda may be considered only as an adjunct to established lifestyle-based management, including weight reduction, dietary modification, exercise, and metabolic risk control. The proposed Ayurvedic contribution includes individualized dietary regulation, support for behavioral adherence, and selected hepatoprotective botanicals such as *Phyllanthus niruri*, *Picrorhiza kurroa*, and *T. cordifolia*; however, these interventions should not be assumed to reverse hepatic steatosis or replace guideline-based cardiometabolic management [26]. Conceptual overlap exists between Ayurvedic ideas of digestive-metabolic imbalance and contemporary models involving the gut-liver axis, inflammation, metabolic endotoxemia, and hepatic lipid accumulation, but Agni and Ama should not be treated as direct substitutes for microbiome, inflammatory, or liver biomarkers [27]. Future studies should use standardized dosing, defined symptom targets, comparator groups, safety monitoring, and objective endpoints such as liver enzymes, imaging-based hepatic fat assessment, fibrosis scores, gut microbiome profiles, inflammatory markers, bowel frequency, stool form, symptom severity, adverse events, and herb-drug interaction assessment. Structured clinical records and Prakriti-linked biomarker profiling may help identify gastrointestinal or hepatobiliary subgroups more likely to respond to diet-based, herbal, or polyherbal interventions.

Applications in musculoskeletal and inflammatory disorders

Ayurveda has been applied as supportive care in musculoskeletal and inflammatory conditions such as low back pain, chronic pain syndromes, osteoarthritis, and rheumatoid arthritis. In Ayurvedic interpretation, pain, stiffness, swelling, tissue degeneration, and functional limitation may be discussed through aggravated Vata, impaired tissue nourishment, accumulation of Ama, and involvement of joints, muscles, and soft tissues [1,7,28]. These concepts should be understood as traditional explanatory models rather than direct equivalents of inflammatory arthritis, cartilage degeneration, autoimmune disease, or nociceptive and neuropathic pain mechanisms. Ayurgenomics may help examine whether Prakriti-based musculoskeletal phenotypes correlate with inflammatory biomarkers, pain sensitivity, immune profiles, metabolic status, cartilage-related markers, or differential response to herbal and procedural interventions [6,7,15].

Ayurvedic management commonly combines internal formulations, external oil therapies, massage, fomentation, dietary regulation, lifestyle correction, rehabilitation, and selected Panchakarma procedures [29]. External procedures such as Abhyanga, Swedana, Janu Basti, and medicated oil application are used with the aim of improving pain, stiffness, mobility, and functional capacity [29,30]. In rheumatoid arthritis-related discussions, Amavata is used as a traditional framework involving Ama, impaired digestive-metabolic function, pain, stiffness, and joint involvement; however, this terminology should not replace modern rheumatologic classification, serological assessment, imaging, or disease-activity scoring. Intervention-specific evaluation should record the formulation or procedure used, treatment duration, co-interventions, analgesic use, baseline disease severity, safety outcomes, and objective functional endpoints.

Botanicals such as *Boswellia serrata*, *Commiphora mukul*, *W. somnifera*, *C. longa*, and *Z. officinale *have been studied for anti-inflammatory, analgesic, antioxidant, and immunomodulatory properties. Curcumin and *C. longa* extracts have been evaluated in arthritis-related conditions, with reported outcomes including pain, stiffness, inflammatory symptoms, physical function, and safety [29]. Turmeric extracts have also been studied in knee osteoarthritis, where clinically relevant endpoints include WOMAC score, pain intensity, stiffness, functional limitation, rescue analgesic use, and adverse events [30]. For low back pain and chronic pain, Ayurvedic approaches may include oil therapy, heat therapy, strengthening exercises, posture correction, and mind-body practices [31,32]. Their potential clinical value lies mainly in adjunctive symptom control, functional rehabilitation, and possible reduction of analgesic dependence where appropriate; however, these effects should be separated from standard rehabilitation, exercise, and lifestyle-based pain management through controlled comparative designs.

**Figure 2:**
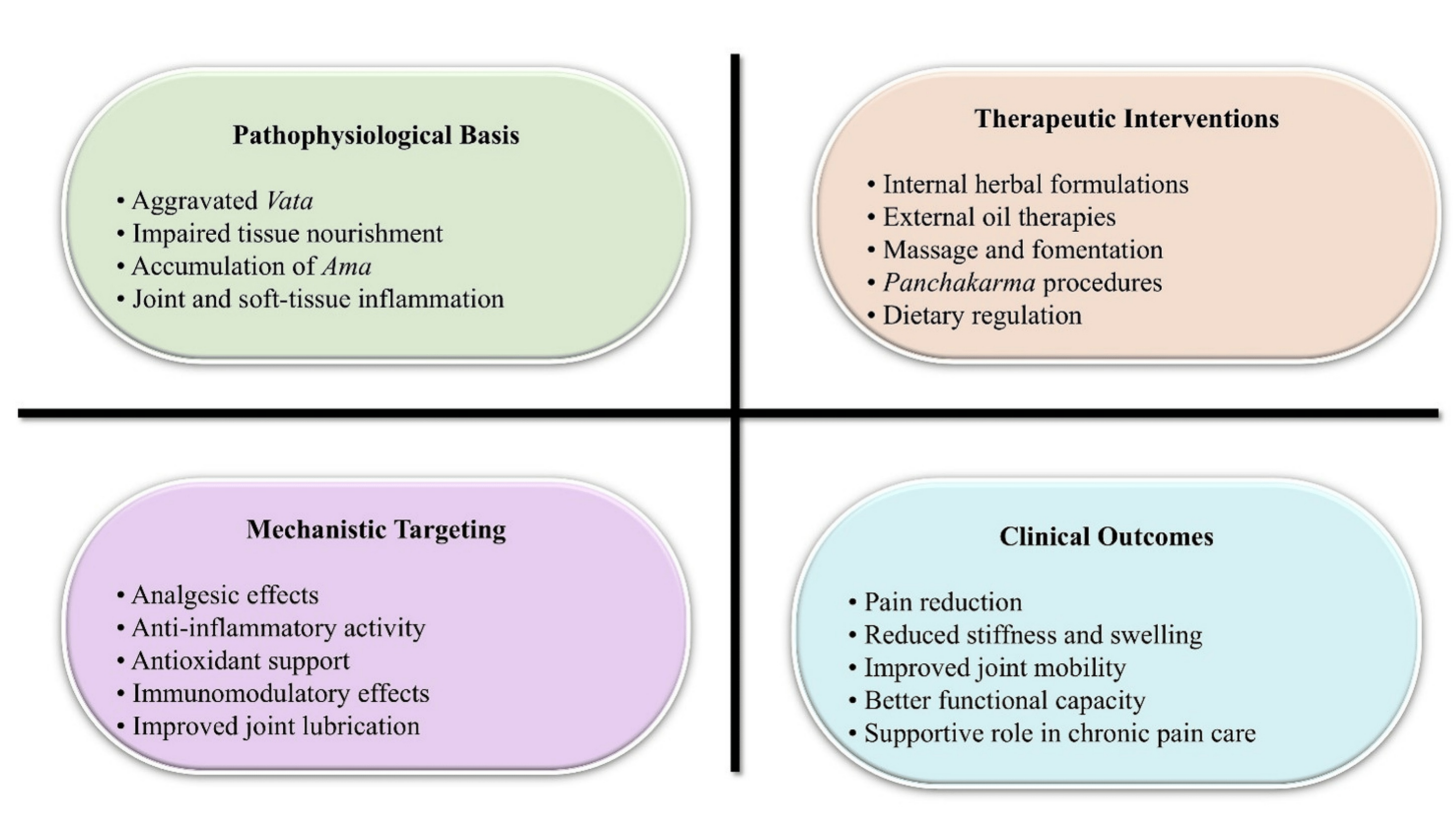
Ayurvedic Management of Musculoskeletal and Inflammatory Disorders Image created by the authors using Microsoft PowerPoint (Microsoft Corp., Redmond, WA, USA).

The current evidence remains limited by small sample sizes, heterogeneous treatment protocols, limited blinding, inconsistent comparator arms, short follow-up, and insufficient long-term safety data [32]. Future studies should use condition-specific endpoints such as visual analogue scale or numerical pain scores, WOMAC scores, DAS28 or other validated disease-activity indices, range of motion, disability scores, analgesic consumption, inflammatory markers, imaging findings, adverse events, and herb-drug interaction monitoring. Structured clinical records should also capture menstrual-cycle data, Prakriti assessment, metabolic biomarkers, reproductive hormone profiles, formulation exposure, dose, adherence, co-medications, pregnancy outcomes, and adverse events to support diagnostic consistency, pharmacovigilance, and response-pattern analysis. Figure 2 summarizes the proposed supportive role of Ayurvedic interventions in musculoskeletal and inflammatory disorders.

Applications in mental health and neurocognitive care

Ayurveda discusses mental health through an integrated model of body regulation, sensory control, digestion, sleep, behavior, and social context [33]. In Ayurvedic literature, disturbances involving Vata, Sattva, Ojas, sleep, and mind-body regulation are used to describe psychological distress, impaired resilience, sleep disturbance, and cognitive or emotional imbalance. These constructs should not be interpreted as direct equivalents of diagnoses defined by the Diagnostic and Statistical Manual of Mental Disorders (DSM) or the International Classification of Diseases (ICD), such as anxiety disorders, depressive disorders, insomnia disorder, or neurocognitive disorders. Their value lies in guiding supportive interventions that can be assessed through validated mental health, sleep, cognitive, stress-biomarker, and quality-of-life outcomes. Ayurgenomics may help examine whether Prakriti-related neurobehavioral phenotypes correlate with stress reactivity, inflammatory markers, neuroendocrine profiles, sleep patterns, or differential response to mind-body and botanical interventions [6,7,15].

Ayurvedic management in this area may include Medhya Rasayana, yoga, meditation, breathing exercises, sleep regulation, dietary correction, daily routine, and behavioral discipline as complementary measures for stress regulation, sleep support, emotional balance, and general well-being [33]. Severe psychiatric, neurocognitive, or sleep disorders require appropriate diagnosis and evidence-based conventional care, with Ayurvedic measures used only as adjunctive support where clinically appropriate. Intervention-specific evaluation should distinguish stress reduction, sleep improvement, and quality-of-life benefits from claims of disease modification in major psychiatric or neurodegenerative disorders.

Medhya Rasayana refers to rejuvenative interventions traditionally used to support memory, intellect, emotional stability, and nervous system function. Botanicals such as *Bacopa monnieri*, *W. somnifera*, *Centella asiatica*, *Nardostachys jatamansi*, and *Convolvulus pluricaulis* have been studied for neuroprotective, anxiolytic, adaptogenic, antioxidant, and cognition-supportive properties [34]. Ashwagandha has been evaluated in randomized and systematic-review evidence, with reported outcomes including perceived stress, anxiety scores, sleep quality, cortisol-related markers, and general well-being [33-36]. Yoga and meditation may support emotional regulation, sleep quality, and resilience [35]. These effects are biologically plausible, but the available evidence remains variable in quality.

Current studies are often limited by short duration, small sample size, subjective outcomes, heterogeneous formulations, inadequate comparator groups, and limited safety monitoring [36]. Future research should use clinically validated endpoints, including standardized anxiety and depression scales, sleep-quality indices, cognitive testing, stress biomarkers, quality-of-life measures, medication use, adverse events, and herb-drug interaction assessment. Structured clinical records should also capture Prakriti assessment, symptom scales, sleep data, formulation exposure, co-medications, patient-reported outcomes, and longitudinal response to distinguish general wellness effects from clinically meaningful improvement. Ayurvedic mental health and neurocognitive interventions should therefore be positioned as supportive strategies for stress regulation, sleep support, and well-being, not as replacements for evidence-based psychiatric, neurological, or sleep-medicine care.

Respiratory and immune-related applications

Ayurveda has traditionally been used for preventive and supportive care for respiratory and immune-related conditions, including asthma, allergic rhinitis, recurrent respiratory infections, and seasonal respiratory symptoms. These conditions are commonly interpreted through imbalance of Kapha and Vata, obstruction of respiratory Srotas, accumulation of Ama, impaired digestive-metabolic function, and reduced Ojas [37]. These constructs may guide Ayurvedic assessment and treatment selection, but they should not be treated as direct equivalents of airway inflammation, bronchial hyperresponsiveness, allergic sensitization, infection susceptibility, or measurable immune dysfunction. Ayurgenomics may help clarify whether Prakriti-based respiratory or immune phenotypes correlate with inflammatory markers, immunoglobulin patterns, airway reactivity, microbiome profiles, seasonal vulnerability, or treatment-response variability [6,7,15].

Ayurvedic respiratory care may include selected herbal formulations, dietary regulation, seasonal regimens, breathing exercises, and Rasayana-based supportive measures [38]. In asthma and allergic rhinitis, the proposed role is symptom support, trigger reduction, improved breathing pattern, and self-management rather than replacement of inhaled bronchodilators, corticosteroids, antihistamines, or emergency respiratory care. Botanicals such as *Adhatoda vasica*, *Glycyrrhiza glabra*, *P. longum*, *Z. officinale*,* Ocimum sanctum*, and *T. cordifolia* have been discussed for bronchodilatory, expectorant, anti-inflammatory, antimicrobial, or immunomodulatory properties, but clinical interpretation requires standardized formulations, dose clarity, comparator groups, and safety monitoring [39]. In allergic rhinitis, Ayurvedic interventions have been evaluated using symptom control and patient-reported improvement, making rhinitis symptom scores, medication use, recurrence, and quality-of-life measures more appropriate than broad claims of “immune enhancement” [39,40].

Breathing practices, especially pranayama, may support respiratory function by improving breathing control, reducing stress-related symptom amplification, and enhancing self-regulation. Rasayana interventions are also used traditionally in recurrent infections and seasonal vulnerability to support Ojas and general resilience [40]. Future studies should evaluate Ayurvedic respiratory and immune-supportive interventions using asthma control scores, allergic rhinitis symptom scores, pulmonary function tests, rescue medication use, exacerbation frequency, infection rates, inflammatory markers, immunoglobulin levels, adverse events, and herb-drug interaction monitoring [38,40]. Structured clinical records should also capture symptom diaries, seasonal exposure, Prakriti assessment, pulmonary function values, formulation details, co-medications, adverse events, and longitudinal outcomes to support diagnostic consistency, pharmacovigilance, and response-pattern analysis. Ayurvedic interventions in this domain should therefore be positioned as preventive or complementary support and not as alternatives to acute, emergency, or guideline-based respiratory care.

Women’s health and reproductive care

Ayurveda describes women’s health through a life-stage and reproductive-health framework that includes menstrual function, metabolic balance, fertility, pregnancy, postnatal recovery, and menopausal transition. In this review, Ayurvedic care is positioned as supportive and adjunctive, not as a replacement for evidence-based gynecologic, obstetric, endocrine, or fertility care. Conditions such as menstrual irregularity, dysmenorrhea, PCOS, fertility-related concerns, menopause-related symptoms, antenatal support, and postnatal recovery are traditionally interpreted through disturbances in Vata, Pitta, Kapha, Agni, Artava dhatu, and reproductive Srotas [41,42]. These concepts may guide individualized Ayurvedic assessment, but they should not be treated as direct substitutes for endocrine, gynecologic, or obstetric diagnoses. Ayurgenomics may help examine whether Prakriti-based reproductive phenotypes correlate with androgen profile, insulin resistance, inflammatory markers, metabolic signatures, menstrual patterns, fertility-related biomarkers, and differential response to lifestyle or botanical interventions [6,7,15].

In PCOS, Ayurvedic management may have relevance only as an adjunct to standard metabolic and reproductive care. Its proposed contribution includes dietary regulation, weight control, physical activity, stress reduction, sleep regulation, menstrual-cycle support, and selected herbal or procedural interventions aimed at improving adherence to long-term lifestyle management. Although the available PCOS literature is not exclusively Ayurveda-specific, lifestyle, movement-based, nutritional, pharmacological, and botanical interventions have been evaluated using outcomes such as body mass index, waist circumference, glycemic indices, lipid profile, androgen profile, menstrual regularity, ovulatory function, and reproductive-metabolic measures [41-44]. Fertility-related support may include individualized diet, lifestyle regulation, stress control, and Rasayana-based measures intended to support reproductive well-being; however, these interventions should not replace ovulation assessment, endocrine evaluation, tubal assessment, semen analysis, assisted reproduction when indicated, or other evidence-based infertility management [43]. During menopause and postnatal recovery, Ayurvedic care may emphasize nutrition, sleep regulation, psychological stability, musculoskeletal support, and restoration of functional strength [44].

The evidence base for Ayurvedic interventions in women’s health remains inconsistent, and broad claims regarding hormonal regulation, fertility improvement, pregnancy safety, or menopause relief should be made cautiously. Future studies should use objective and clinically relevant endpoints, including menstrual-cycle regularity, ovulation status, androgen profile, insulin resistance markers, body mass index, waist circumference, fertility outcomes, pregnancy safety, menopausal symptom scores, postnatal recovery measures, quality of life, adverse events, and herb-drug interaction monitoring. Artificial intelligence and machine learning may strengthen this field by digitizing menstrual-cycle records, Prakriti assessment, metabolic biomarkers, reproductive hormone profiles, formulation exposure, dose, adherence, co-medications, pregnancy outcomes, and adverse events [45,46]. These structured datasets may support diagnostic standardization, pharmacovigilance, and prediction of response to individualized or polyherbal therapy in PCOS and other reproductive-health contexts. When clinical risk is present, Ayurvedic measures should be used only as complementary support and should not replace evidence-based obstetric, gynecologic, endocrine, or fertility care. The therapeutic use of Ayurveda in various major clinical fields has been summarized in Table 2.

**Table 2:** Therapeutic Applications of Ayurveda Across Major Clinical Domains HbA1c: hemoglobin A1c; IBS: irritable bowel syndrome; WOMAC: Western Ontario and McMaster Universities Osteoarthritis Index; DAS28: Disease Activity Score in 28 joints; AI: artificial intelligence; PCOS: polycystic ovary syndrome; BMI: body mass index; ADL: activities of daily living

Clinical Domain	Specific Interventions	Reported/Measurable Outcomes	Critical Interpretation	References
Metabolic and cardiovascular disorders	Ayurvedic herbal preparations, *Tinospora cordifolia*, lifestyle regulation, yoga, dietary correction, Prakriti-guided cardiometabolic risk assessment	Lipid profile, triglycerides, fasting glucose, HbA1c, body weight, blood pressure, waist circumference, adherence, medication use, and adverse events	Supportive role possible; formulation variability and trial heterogeneity limit definitive claims; Ayurgenomics may help link Prakriti, Meda-related phenotypes, lipid metabolism, inflammatory markers, and cardiometabolic risk profiles	[6,7,10,19,22,23]
Gastrointestinal disorders	Whole-system Ayurveda, digestive herbs, carminatives, Triphala, bowel routine correction, Agni-Ama assessment with structured dietary and symptom recording	IBS symptom severity, abdominal pain, bloating, bowel frequency, stool form, quality of life, dietary triggers, rescue medication use, and adverse events	Useful only as supportive care after appropriate diagnostic evaluation; microbiome and metabolomic profiling may help validate Agni- and Ama-related gastrointestinal phenotypes	[24-28,45]
Musculoskeletal and inflammatory disorders	Curcumin, turmeric extracts, anti-inflammatory herbs, oil therapies, Panchakarma-related procedures, Prakriti-stratified pain and inflammation assessment	Pain score, WOMAC, stiffness, DAS28, function, range of motion, analgesic use, inflammatory markers, imaging findings, and adverse events	Evidence is strongest for selected botanicals; whole-system protocols need stronger testing; machine learning may help predict response to polyherbal or procedural interventions using pain, biomarker, imaging, Prakriti, and treatment-composition data	[6,7,15,29-32]
Mental health and stress-related care	Ashwagandha, Medhya Rasayana, yoga, meditation, sleep regulation, digitized stress, and sleep monitoring	Perceived stress, anxiety scores, sleep quality, cortisol-related markers, quality of life, patient-reported outcomes, medication use, and adverse events	Adjunctive stress-support role; not a replacement for psychiatric care; AI-supported longitudinal symptom tracking may help distinguish general wellness effects from clinically meaningful improvement	[33-36]
Respiratory and immune-related care	Ayurvedic interventions for allergic rhinitis, pranayama, Rasayana, respiratory herbs, seasonal regimen, and exposure tracking	Rhinitis symptom score, asthma control, pulmonary function, rescue medication use, exacerbations, infection frequency, inflammatory markers, immunoglobulin levels, and adverse events	“Immunity” claims require objective respiratory and biomarker endpoints; AI-assisted pharmacovigilance may identify herb-drug interactions and safety signals in patients using respiratory medications	[37-40]
Women’s health and PCOS-related care	Lifestyle regulation, dietary intervention, selected botanical or supportive approaches, and Prakriti-linked reproductive and metabolic profiling	Menstrual regularity, ovulation status, androgen profile, insulin resistance, BMI, lipid profile, waist circumference, fertility outcomes, pregnancy safety, and quality of life	Lifestyle relevance is clear; Ayurveda-specific claims need direct trials; machine learning may help predict response to individualized or polyherbal therapy using menstrual, hormonal, metabolic, Prakriti, dose, and adherence data	[6,7,15,41-44]
Geriatric and healthy-aging care	Rasayana, nutrition, sleep regulation, gentle activity, rehabilitation support, digital functional monitoring	Frailty index, gait speed, grip strength, cognition, ADL score, fall risk, quality of life, hospitalization, medication burden, inflammatory markers, and adverse events	Rejuvenation claims require measurable functional and safety outcomes; biomarker-linked aging profiles and AI-based longitudinal records may support safer evaluation of Rasayana and polyherbal interventions	[18,20,47]

Geriatric care and healthy aging

Geriatric care should be discussed separately because its clinical priorities differ from those of women’s health and reproductive care. In older adults, Ayurveda is mainly relevant as supportive care for healthy aging, functional maintenance, recovery, resilience, sleep regulation, appetite, mobility, cognition, and quality of life. Rasayana therapy is traditionally used to support vitality, tissue nourishment, immune resilience, and long-term well-being [18,20,47]. These claims should be interpreted cautiously because healthy aging is multidimensional and cannot be established through general wellness descriptions alone. Ayurgenomics may help evaluate whether Prakriti-based aging patterns correlate with genomic susceptibility, inflammatory markers, metabolic status, frailty phenotypes, cognitive reserve, immune function, and response to Rasayana or polyherbal interventions [6,7,15].

The possible contribution of Ayurvedic geriatric care may lie in individualized lifestyle regulation, nutrition, sleep hygiene, gentle physical activity, selected Rasayana formulations, and supportive rehabilitation. These interventions may be useful when integrated with conventional geriatric assessment, medication review, fall-risk evaluation, chronic disease management, and functional monitoring. Specific outcomes should include frailty indices, activities of daily living, gait speed, grip strength, cognition, sleep quality, appetite, nutritional status, fall risk, hospitalization, medication burden, inflammatory markers, quality of life, and adverse events. Ayurvedic interventions should not replace evidence-based geriatric diagnosis or treatment, particularly in frailty, dementia, polypharmacy, cardiovascular disease, diabetes, osteoporosis, infection, or acute illness.

Future research should evaluate Ayurvedic geriatric interventions using standardized outcomes, including frailty indices, activities of daily living, gait speed, grip strength, cognition, sleep quality, inflammatory markers, nutritional status, medication burden, adverse events, herb-drug interactions, fall risk, hospitalization, and quality-of-life measures. Structured clinical records should also capture Prakriti assessment, Rasayana exposure, polyherbal composition, comorbidities, co-medications, laboratory safety data, falls, hospitalization, patient-reported outcomes, and longitudinal functional response to support geriatric assessment, pharmacovigilance, and response-pattern analysis. Stronger evidence is needed before claims related to “rejuvenation,” longevity, immune resilience, or cognitive protection can be translated into clinical recommendations.

Panchakarma, Rasayana, and lifestyle medicine

Panchakarma, Rasayana, and Ayurvedic lifestyle medicine are important components of preventive and supportive care, but their clinical interpretation requires careful distinction between traditional rationale and evidence-based therapeutic claims. Panchakarma is traditionally described as a purification and restorative approach intended to remove accumulated imbalance, support digestive-metabolic function, and restore physiological stability. It includes procedures such as Vamana, Virechana, Basti, Nasya, and Raktamokshana [45]. In contemporary integrative settings, Panchakarma has been used in metabolic disorders, musculoskeletal pain, inflammatory conditions, stress-related symptoms, and general wellness; however, protocols vary substantially across practitioners and institutions, limiting reproducibility and comparison across studies [46]. Its clinical value should therefore be evaluated through standardized procedural reporting, objective endpoints, adverse-event monitoring, and clearly defined comparator groups rather than through broad claims of detoxification or restoration. For metabolic or musculoskeletal indications, measurable outcomes should include HbA1c, lipid profile, body mass index, pain scores, WOMAC score, range of motion, inflammatory markers, functional capacity, medication use, and adverse events.

Rasayana therapy is traditionally directed toward tissue nourishment, vitality, resilience, healthy aging, cognitive support, and long-term well-being. It may include herbal formulations, dietary regulation, behavioral discipline, sleep management, and measures intended to support Ojas [47]. Commonly used Rasayana botanicals, including *W. somnifera*, *E. officinalis*, *T. cordifolia*, *B. monnieri*, and *Asparagus racemosus*, have been studied for antioxidant, immunomodulatory, adaptogenic, neuroprotective, and anti-inflammatory properties [47]. These findings provide biological plausibility, but they do not automatically establish clinical efficacy. Future studies should define formulation composition, dose, duration, target population, safety profile, and measurable outcomes such as fatigue, cognition, frailty, inflammatory markers, quality of life, and adverse events. Ayurgenomics may strengthen Rasayana research by examining whether Prakriti-based aging, immune, cognitive, or resilience phenotypes correlate with genomic profiles, inflammatory signatures, metabolomic markers, frailty measures, and differential responses to Rasayana or polyherbal formulations [6,7,15].

Ayurvedic lifestyle medicine includes Dinacharya or daily routine, Ritucharya or seasonal regimen, dietary discipline, physical activity, yoga, behavioral regulation, and sleep hygiene. These practices overlap with contemporary preventive medicine because they target modifiable determinants of chronic disease, including physical inactivity, unhealthy diet, psychological stress, sleep disturbance, and irregular daily habits [45]. The possible added value of Ayurveda lies in individualized lifestyle structuring and long-term adherence support; however, its independent effect beyond general lifestyle medicine remains difficult to determine without comparative studies. Future research should assess adherence, metabolic outcomes, sleep quality, stress measures, functional status, quality of life, adverse events, and integration with standard medical care. Structured clinical records should also capture daily routine, seasonal regimen, diet, yoga practice, sleep, Prakriti, formulation exposure, adherence, clinical outcomes, and safety events to improve reproducibility and response-pattern analysis. Beyond intervention-level standardization, the modernization of Ayurveda also requires digital and molecular approaches that can convert traditional phenotyping, clinical documentation, and treatment response into reproducible and measurable datasets. The key Ayurvedic interventions, their proposed roles, limitations, and research priorities are presented in Table 3.

**Table 3:** Key Ayurvedic Interventions, Limitations, and Future Research Priorities

Intervention Category	Main Components	Proposed Role in Modern Healthcare	Current Limitations	Future Research Priority	Reference
Herbal formulations	Single herbs and polyherbal combinations such as *Withania somnifera*, *Curcuma longa*,* Tinospora cordifolia*, *Boswellia serrata*, and *Bacopa monnieri*	Supportive management of inflammation, metabolism, cognition, immunity, and chronic symptoms; potential use in disease-specific outcomes such as lipid profile, pain scores, stress measures, and gastrointestinal symptoms	Variation in formulation, dosage, raw material quality, active constituents, batch consistency, and herb-drug interaction reporting	Standardized formulations, phytochemical profiling, dose-response studies, safety monitoring, AI-assisted prediction of response to polyherbal therapy using Prakriti, biomarkers, dose, co-medications, and outcomes	[20,23]
Panchakarma	Vamana, Virechana, Basti, Nasya, Raktamokshana, oil therapies, fomentation	Potential role in chronic pain, metabolic dysfunction, inflammatory disorders, and restorative care; requires indication-specific assessment rather than broad claims of detoxification	Protocol heterogeneity, practitioner dependence, unclear biomedical endpoints, and limited reproducibility across centres	Pragmatic trials, standardized procedural reporting, objective biomarkers, adverse-event documentation, digital recording of procedure type, duration, sequence, patient phenotype, and clinical response	[29,46]
Rasayana therapy	Rejuvenative herbs, diet, behavioral discipline, sleep regulation, and vitality-promoting regimens	Healthy ageing, immune resilience, cognitive support, recovery, and preventive care; potential supportive role in frailty, fatigue, cognition, sleep, and quality-of-life outcomes	Broad claims related to rejuvenation and immunity are often insufficiently operationalized; limited biomarker-linked evidence for resilience, aging, and immune function	Functional ageing outcomes, immune biomarkers, cognitive testing, quality-of-life measures, Ayurgenomics-based mapping of Prakriti to molecular ageing, inflammatory signatures, and Rasayana response	[18,20]
Ayurvedic lifestyle medicine	Dinacharya, Ritucharya, dietary regulation, yoga, meditation, sleep hygiene, exercise	Prevention and long-term management of lifestyle-related disorders may support adherence, cardiometabolic risk reduction, stress regulation, sleep improvement, and patient self-management	Difficult to separate the effects of diet, exercise, counselling, yoga, and behavioral adherence; limited comparative evidence against standard lifestyle medicine	Comparative effectiveness studies, adherence measures, metabolic and psychosocial outcomes, digital tracking of diet, sleep, activity, seasonal routines, Prakriti, and longitudinal clinical outcomes	[22,47]
Mind-body practices	Yoga, pranayama, meditation, behavioral regulation, sensory control	Stress reduction, sleep improvement, respiratory support, and mental health support; potential measurable benefits in anxiety scores, perceived stress, sleep quality, breathing control, and quality of life	Variable intervention duration, intensity, outcome assessment, and limited standardization of practice protocols	Standardized intervention manuals, validated psychiatric and respiratory outcomes, wearable and digital patient-reported data to monitor adherence, physiologic response, and safety	[33,38]
Integrative care models	Combined Ayurvedic and biomedical assessment, risk stratification, monitoring, and coordinated care	Complementary support in chronic disease, rehabilitation, prevention, and quality-of-life improvement may enable coordinated use of Ayurveda with conventional care when safety monitoring is explicit	Limited clinical guidelines, uneven professional collaboration, risk of unsupervised use, inconsistent diagnostic documentation, and weak pharmacovigilance systems	Interdisciplinary protocols, safety frameworks, referral criteria, real-world evidence studies, AI-supported clinical records, diagnostic standardization, adverse-event detection, and polyherbal response prediction	[3,47]

Ayurgenomics, artificial intelligence, and digital standardization

Ayurgenomics is an emerging translational field that may help bridge classical Ayurvedic phenotyping with contemporary precision medicine. Its central premise is that Prakriti-based constitutional classification may correspond to measurable biological variation in genomic, transcriptomic, epigenomic, metabolomic, microbiome, inflammatory, immune, and metabolic profiles [6,7,15]. This approach does not require treating Vata, Pitta, and Kapha as direct biomedical entities. Rather, it allows Prakriti to be studied as a structured phenotypic framework that may be mapped against molecular signatures, disease susceptibility, preventive needs, and treatment-response patterns [6]. Such mapping may strengthen individualized Ayurveda by replacing broad constitutional descriptions with testable biological associations.

Ayurgenomics may support clinical translation by improving phenotypic stratification, preventive risk assessment, and clinical trial design. It may help identify biological differences among patients who share the same biomedical diagnosis but differ in constitution, metabolic pattern, inflammatory status, stress response, or treatment tolerance [6,7]. It may also link Prakriti categories with measurable risk markers such as lipid profile, glucose regulation, cytokine patterns, autonomic function, gut microbiome features, or metabolomic signatures [6,7,27]. In clinical trials, Prakriti-based subgrouping may reduce heterogeneity and clarify whether specific Ayurvedic formulations or lifestyle interventions work better in defined constitutional or molecular subgroups [8,15,17]. This is relevant because Ayurvedic interventions are commonly individualized, multimodal, and difficult to evaluate using conventional uniform-treatment trial models [14,17].

Artificial intelligence and machine learning may further modernize Ayurveda by improving documentation, diagnostic consistency, outcome tracking, pharmacovigilance, quality control, and therapeutic prediction. Digitized Ayurvedic records can convert narrative clinical assessments into structured datasets that include Prakriti, Vikriti, Agni, Ama, Srotas involvement, symptoms, diet, lifestyle factors, formulations, Panchakarma procedures, dose, duration, co-medications, laboratory parameters, adverse events, and clinical outcomes [8,12,15]. These datasets may support diagnostic standardization, reduce inter-practitioner variability, and help predict response to polyherbal therapy by integrating traditional variables with biomedical parameters such as laboratory markers, imaging findings, microbiome data, medication history, comorbidities, and patient-reported outcomes [4,6,8].

Artificial intelligence may also strengthen pharmacovigilance and quality control. Natural language processing can extract adverse events from clinical notes, patient reports, and electronic health records, while predictive models may identify patients at increased risk of herb-drug interactions, hepatotoxicity, nephrotoxicity, heavy metal exposure, adulteration-related harm, dose-related toxicity, or poor adherence [9,12]. Machine learning can also connect botanical authentication, phytochemical fingerprints, contaminant testing, batch variability, formulation composition, and clinical outcomes, which is particularly important for polyherbal therapy because multiple active constituents may interact with several biological pathways and conventional medications [9,20].

The combined use of Ayurgenomics, artificial intelligence, and machine learning should be viewed as a modernization strategy rather than proof of efficacy. These tools may improve phenotyping, documentation, standardization, response prediction, pharmacovigilance, quality control, and trial design, but they cannot replace rigorous clinical validation [14,17]. Their reliability will depend on large multi-centre datasets, standardized data elements, transparent algorithms, external validation, ethical data governance, explainability, and integration with regulated clinical practice [4,8,15]. Under these conditions, digital and molecular approaches may help convert individualized Ayurvedic practice into a more testable, reproducible, and safety-monitored model of preventive and supportive healthcare (Table 4).

**Table 4:** Digital and Molecular Approaches for Modernizing Ayurveda AI: artificial intelligence; HbA1c: hemoglobin A1c; IBS: irritable bowel syndrome

Approach	Application	Expected Contribution	Key Requirement	References
Ayurgenomics	Mapping Prakriti to genomic, transcriptomic, metabolomic, immune, and microbiome profiles	Bridges classical constitutional assessment with precision medicine; links Prakriti body types with molecular biomarkers, disease susceptibility, and treatment-response patterns	Large validated cohorts, reproducible molecular signatures, Prakriti-stratified clinical datasets, and external validation	[6,7,15]
Biomarker profiling	Cytokines, lipid profile, glycemic markers, microbiome, metabolomics, autonomic and endocrine markers	Converts traditional assessment into measurable biological domains; supports objective evaluation of Agni, Ama, Ojas, metabolic risk, inflammation, stress response, and functional recovery	Standardized sampling, validated assays, longitudinal follow-up, and disease-specific endpoints such as HbA1c, lipid profile, IBS scores, pain scores, hormone profile, frailty indices, and quality-of-life measures	[6,7,27]
AI-based clinical records	Digitized Prakriti, Agni, Ama, symptoms, prescriptions, outcomes, adverse events, formulation composition, dose, duration, co-medications, and adherence	Improves documentation, audit, research-quality data capture, diagnostic reproducibility, inter-practitioner consistency, and longitudinal outcome tracking	Structured electronic data fields, interoperable databases, standardized Ayurvedic diagnostic criteria, data governance, and routine adverse-event recording	[4,8,15]
Machine learning prediction	Prediction of response to polyherbal therapy using clinical, Prakriti, laboratory, and safety variables	Supports individualized treatment selection and subgroup analysis; may identify predictors of response in metabolic, gastrointestinal, musculoskeletal, mental health, respiratory, reproductive, and geriatric conditions	Transparent models, external validation, explainability, large multicentre datasets, predefined outcomes, and independent testing cohorts	[6,8,17]
AI-assisted pharmacovigilance	Detection of adverse events, herb-drug interactions, toxicity signals, and high-risk patient groups	Strengthens safety monitoring in integrative care; supports early detection of hepatotoxicity, nephrotoxicity, heavy metal exposure, adulteration-related harm, dose-related toxicity, and interaction risk	Linked clinical, laboratory, prescription, and adverse-event datasets; active surveillance systems and standardized toxicity-reporting protocols	[9,12]
Quality-control analytics	Botanical authentication, phytochemical fingerprinting, contaminant testing, batch comparison, and linkage of batch-level data with clinical outcomes	Reduces formulation variability, adulteration, and safety risk; improves reproducibility of polyherbal interventions and interpretation of therapeutic outcomes	Regulatory-grade testing, batch-level traceability, phytochemical standardization, contaminant thresholds, and integration with clinical response and safety datasets	[9,20]

Limitations and future directions

The current evidence base is limited by heterogeneity across Ayurvedic interventions, study designs, diagnostic approaches, formulations, dosage regimens, treatment duration, practitioner expertise, comparator groups, and outcome measures. Many studies remain small-scale and are affected by unclear randomization, limited blinding, short follow-up, incomplete dropout reporting, and insufficient adverse-event documentation. These limitations reduce reproducibility and restrict translation into routine clinical recommendations. Standard trial models are also difficult to apply without adaptation because Ayurvedic care often involves individualized assessment using Prakriti, Agni, Ama, Dhatu, Srotas, and Ojas, along with multimodal interventions combining diet, lifestyle, herbal formulations, procedures, and behavioral regulation. Future studies should reduce this heterogeneity by reporting disease-specific interventions and outcomes, including lipid profile and HbA1c in metabolic disease, IBS symptom severity and stool form in gastrointestinal disorders, WOMAC and DAS28 in musculoskeletal disease, stress and sleep scales in mental health care, pulmonary function and rhinitis scores in respiratory care, reproductive-metabolic parameters in PCOS, and frailty or activities-of-daily-living measures in geriatric care.

Safety and quality-control concerns remain major barriers to clinical integration. Product variability, inconsistent botanical authentication, uncertain active-constituent profiles, contamination, heavy metal exposure, adulteration, dose inconsistency, herb-drug interactions, and inadequate pharmacovigilance may affect both efficacy and patient safety. These concerns are especially important when Ayurvedic formulations are used alongside conventional therapies in patients with chronic diseases, polypharmacy, pregnancy, older age, renal or hepatic impairment, or immune compromise. Future studies should include systematic toxicity monitoring, adverse-event reporting, laboratory safety parameters, herb-drug interaction assessment, and documentation of manufacturing quality. Digital pharmacovigilance may further support safety monitoring by linking herb exposure, laboratory markers, co-medications, adverse events, and batch-level formulation data.

Future research should prioritize well-designed randomized controlled trials, pragmatic clinical studies, real-world evidence, and long-term safety surveillance. Intervention reporting should include formulation composition, source authentication, dosage, duration, procedural details, practitioner qualifications, adherence assessment, comparator selection, and co-interventions. Outcome assessment should move beyond general wellness descriptions and include validated clinical endpoints, biomarkers, patient-reported outcomes, functional measures, medication use, quality of life, and adverse events. Ayurgenomics, standardized digital records, and machine-learning models may strengthen future research by mapping Prakriti to molecular and clinical phenotypes, improving diagnostic consistency, supporting biomarker-linked subgrouping, and predicting response to polyherbal therapy. Systems biology, pharmacology, genomics, metabolomics, microbiome studies, and digital health tools may strengthen mechanistic and translational evaluation, but these approaches should complement rather than replace rigorous clinical validation. Evidence-based clinical guidelines, interdisciplinary collaboration, quality-controlled manufacturing, pharmacovigilance systems, external validation of predictive models, and ethical governance of digital datasets are essential for safe, ethical, and clinically meaningful integration of Ayurveda into contemporary healthcare.

## Conclusions

This review indicates that Ayurveda may contribute to contemporary healthcare mainly as a preventive, supportive, and lifestyle-oriented system rather than as a stand-alone substitute for conventional diagnosis or treatment. Its potential relevance lies in structured lifestyle regulation, individualized care, chronic disease support, rehabilitation, women’s health, healthy aging, and patient-centred health promotion. Specific interventions discussed in this review include Ayurvedic herbal preparations for dyslipidemia and hypertriglyceridemia, whole-system Ayurveda for IBS, curcumin and turmeric extracts for arthritis and knee osteoarthritis, Ashwagandha for stress and anxiety, Ayurvedic interventions for allergic rhinitis, lifestyle-based approaches for PCOS-related metabolic outcomes, Rasayana-based geriatric support, and Panchakarma-related procedural care. Reported or measurable outcomes include lipid profile, HbA1c, gastrointestinal symptom scores, WOMAC and pain scores, anxiety and sleep measures, respiratory symptom scores, reproductive-metabolic parameters, frailty indices, and quality-of-life measures. Core Ayurvedic concepts such as Prakriti, Agni, Ama, Ojas, Panchakarma, and Rasayana may provide useful traditional frameworks for understanding constitution, digestion-metabolism, resilience, restoration, and long-term well-being; however, these constructs should not be treated as direct biomedical equivalents without empirical validation. The available evidence remains heterogeneous, and translational confidence is limited by small sample sizes, variable formulations, inconsistent protocols, short follow-up, limited comparator groups, and inadequate adverse-event reporting. Safety also requires greater attention, particularly regarding herb-drug interactions, contamination, heavy metal exposure, dose standardization, and pharmacovigilance. Emerging Ayurgenomics may help bridge this translational gap by mapping Prakriti-based body types to genomic profiles, molecular biomarkers, metabolic signatures, inflammatory markers, microbiome patterns, and treatment-response phenotypes. Artificial intelligence and machine learning may further support modernization by digitizing Ayurvedic clinical records, standardizing diagnostic criteria, improving pharmacovigilance, linking formulation quality with clinical outcomes, and predicting individualized response to polyherbal therapy. Future integration should depend on rigorous clinical trials, validated biomarkers, quality-controlled formulations, transparent toxicity monitoring, interdisciplinary care models, structured digital datasets, externally validated predictive models, and reproducible molecular-clinical correlations. Under these conditions, Ayurveda may serve as a complementary approach for prevention, lifestyle regulation, rehabilitation, and patient-centred supportive care.
